# Parental Licensing as Harm Reduction

**DOI:** 10.1007/s10728-020-00404-y

**Published:** 2020-10-17

**Authors:** Liam Shields

**Affiliations:** University of Manchester, 4.027, Arthur Lewis Building, Oxford Road, Manchester, M139PL UK

**Keywords:** Harm reduction, Parental licensing, Parents’ rights, Children’s welfare

## Abstract

In this paper, I will argue that some prominent objections to parental licensing rely on dubious claims about the existence of a very stringent, if not indefeasible, right to parent, which would be violated by licensing. I claim that attaching such stringency to the right only makes sense if we make a number of idealising assumptions. Otherwise, it is deeply implausible. Instead, I argue that we should evaluate parental licensing policies in much the same way we would harm reduction policies. By adopting this critical perspective, we can see that there are powerful, but quite different, reasons to be cautious about parental licensing relating to our ability to minimize the harmful effects of mass-parenting in a world of minimal surveillance and intervention.

## Introduction

Advocates of parental licensing believe that competency in parenting should be demonstrated prior to the allocation of full legal parental rights.^1^ These rights generally include permissions to exclude others from interacting with the child, to decide where the child lives and is schooled, and other rights that it is necessary for a guardian to have in order to meet the child’s needs. However, parents’ legal rights and social status provide them with opportunities to abuse, neglect or otherwise seriously harm the children in their care. In many contemporary societies, surveillance measures and checks on parental competence are used at various stages, in particular by health and education professionals, but that surveillance and intervention is kept to a minimum. The putative justifying aim of parental licensing is to minimize parental child abuse and inadequate parenting consistent with the status quo of limited surveillance and intervention in the family.^2^ After all, there are good reasons for maintaining limited surveillance: parenting well requires a measure of discretion, authority and intimacy all of which are arguably inconsistent with very close surveillance from agents who can override parental decisions.

In this paper, I will argue that some prominent objections to parental licensing rely on dubious claims about the existence of a very stringent right to parent, which would be violated by licensing. I claim that attaching such stringency to the right only makes sense if we make a number of idealising assumptions. But since the philosophy of parental licensing is an exercise in non-ideal theory, this level of idealisation is in tension with what motivates the proposal in the first place: to help address child abuse in our currently unjust world. In this context, a commitment to such stringent rights to parent cannot be maintained. I argue that our normative philosophical evaluation of parental licensing proposals should proceed in much the same way as it does for harm reduction policies. By adopting this critical perspective, we can see that there are powerful, but quite different, reasons to be cautious about parental licensing relating to its likely administration.

## I

In 1980, Hugh LaFollette offered the original argument for parental licensing. He argues from analogy with other licensed activities, such as driving, gun ownership and the medical and legal professions (1980: 183).^3^ If restrictions on liberty for these activities are justified then so must they be for parenting too.

Exemplifying one type of objection to parental licensing, S. Matthew Liao argues, in his *The Right to be Loved,* that all people have a human right to become biological parents and that licensing would interfere with that right by putting an obstacle in the way of it: the competence test.^4^ As such, licensing would violate our human right to become biological parents and is unjustified. Liao believes that adults acquire very stringent rights to parent their procreative children automatically. If Liao is correct that parental rights arise automatically and are not conditional on *proven* competence, then they would be violated.^5^ It seems sensible for the critic of licensing, like Liao, to accept that those who would be incompetent parents do not have such a right automatically, especially those who have previously harmed, abuse or neglected children in their care. However, for those parents who would do a good job, this right would be violated by a licensing system.

To deny that parental rights can arise automatically even for competent parents, would be to say that no adult has rights over any child until competence is proven, via licensing. The truth of such a claim would make justifying licensing easier, but it is not plausible because it implies that under our current system of no licensing no one has a right to parent their children. The violation of competent parents’ rights is what underlies the objection to parental licensing.

Another objection appears to have a more pragmatic basis, but, as I will show, it relies on the very same normative claim as the stringent rights objection. Michael Wald and Michael Sandmire have argued, in response to Claudia Mangel’s licensing proposals, that procedures for determining parental competence face significant practical difficulties, particularly with respect to the accuracy of competency testing.^6^ While noting several problems with testing, Wald and Sandmire’s analysis emphasises that the tests we have for parental competency are seriously ‘Inaccurate on False Negatives’. This means that the group of adults who fail the test will contain very many competent parents and who should have passed it. This inaccuracy is due to the low incidence of child abuse in the population in general and so can be expected to apply to any licensing system.^7^ It is not merely a feature of current technology that may be overcome. Parental licensing therefore denies many good parents a license. If, as Wald and Sandmire suggest, the number of good parents denied a license will be very large, then there will be a very large number of rights violations. But this problem, although seemingly to do with accuracy of tests, is only as big a problem as the rights violations themselves.^8^ The normative force of inaccuracy, in other words, is derived from the violation of the rights of would-be parents.

And so we can see that both of these objections to parental licensing rely not only on the existence of parental rights, which is relatively uncontroversial, but also on the premise that rights are hard constraints on policy, or at least cannot be overridden by the prospective reduction in the incidence of child abuse and neglect. Indeed, Wald and Sandmire do note that tests can identify potential abusers as such and there will be a trade-off between accuracy in false negatives and accuracy in false positives. In their words, “the broader one casts one's nets to catch abusers, the more likely one is to catch all of them but at a cost of catching many other people who will not be abusers.”^9^ That parental rights are sufficiently stringent to reject licensing, then, is the issue we must confront.

In order to understand the stringency of the right to parent we must ask what interest(s) underpins it.^10^ Presumably it is an interest in parenting and not in seeing a child as the property of their biological parents. The most persuasive characterization of a weighty parental interest comes from Brighouse and Swift, who claim that what is wrong with raising all children in orphanages is that it would “leave perfectly competent parents missing out on the goods of parenting”.^11^ A similar consequence follows from licensing where the tests for competence are even slightly inaccurate on false negatives. Brighouse and Swift argue that parenting is highly and uniquely valuable.^12^ It makes a non-substitutable contribution to the well-being of many adults to parent well. These people cannot fully flourish without it. The greater the number of persons this is true for the stronger the argument against licensing.

However, if licensing is moderately accurate on false positives, i.e. it would lead to fewer abusers being given the opportunity to abuse than without licensing, then it diminishes the risk of children being abused. Even if the number of children who would be abused without licensing was much smaller than the number of adults who would be deprived of the means of their fully flourishing by licensing, it would be unjustifiable to not have licensing due to the relative importance of the interests at stake.

This situation now resembles transmitter room cases whereby a large number of individuals enjoy a lesser benefit at the expense of a smaller number or a single individual incurring a very large cost.^13^ The interest that children have in avoiding abuse, and the very stringent right they have against it, cannot be easily traded off for gains in terms of adult’s fully flourishing. Indeed, the category of things one needs to fully flourish does not seem all that weighty a category. There are many things one cannot fully flourish without that one does not have a right to, but even if it were quite weighty it is not plausibly weightier than the interest children have against being abused. The same very substantial burden of argument will push back against other characterizations of the parental interest that grounds the right to parent. For this reason, it seems that any moderately accurate licensing scheme that identifies parents who would otherwise have a high risk of abusing their children can be justified by helping to avoid abuse which overrides the importance of the parental interest which typically grounds a right.

In order to undermine justifications for parental licensing, parents would have to have a very stringent right to parent. I submit that the right to parent is simply not that stringent. It cannot support a rejection of all licensing policies that would reduce child abuse by denying competent parents the opportunity to do so. This is because the rights of children not to be victims of child abuse is much stronger than any permission to parent a child, biological or otherwise, even assuming a weighty interest in parenting, such as the one that Brighouse and Swift articulate.^14^

Some critics will baulk at the suggestion that rights can be traded-off in this way. But to proceed as if parental rights are absolute constraints on licensing policies is a mistake, not least because, if we insist that rights are hard constraints, there is an irresolvable rights conflict between the rights of parents and of children in this case.

With some idealising assumptions, the argument from parental rights against parental licensing can be made to work. If there is a non-rights violating alternative that reduces child abuse no less than licensing, then the violation of parental rights will be decisive. Also, if our tests were 100% accurate we would never deprive good parents, those who it is most plausible to think have such a right, of the opportunity to parent.^15^ But those assumptions are not warranted for the only salient normative examination of parental licensing. Parental licensing is a response to the inevitability of child abuse and neglect due to parental failures. In a perfectly just world, there would be no parental licensing because the rights of parents and the rights of children would be in harmony. No parent, or other, would perpetrate child abuse. But child abuse and neglect will continue. What we have to do is decide between the instruments that can reduce the rate of maltreatment. Those instruments may involve rights infringements or violations, and our job in taking parental licensing proposals seriously is not to imagine possible objections from the perspective of a world free from rights violations, but rather to navigate a set of likely circumstances where at least some rights violations are a fixed part. So, it is not just that arguments from the stringent right to parent are implausible, they also take the wrong approach and obscure the many other relevant factors in determining whether and when parental licensing would be justified.

## III

Harm reduction is an approach to policy evaluation that is most familiar from policies regarding drug use. Harm reduction initiatives have included the provision of needle exchanges, mentorship, supervised consumption space, and substitutes for certain drugs. The harm reduction approach is characterised by an acceptance of certain undesirable practices that often pose risks to health and well-being of those engaged in these practices and third parties, while attempting to minimize the bad effects of those practices, while they continue. The acceptance of the undesirable practice means that policies of harm reduction do not aim to completely stop that practice at any cost, nor do they call for harsh crack-down on the practices through criminal or legal routes either because it would be too morally costly or practically infeasible. Rather harm reduction aims to try to improve things for those affected, so that the undesirable consequences are mitigated.

With respect to licensing parents, the aim of the policy is to make parenting safer for children and, in particular, to diminish the incidence of the severe harms of parental child abuse and neglect. We know that, sad as it may be, entirely eradicating child abuse and neglect is practically impossible. Very heavy surveillance could be adopted, but this would likely sacrifice opportunities for intimacy between all parents and children, the very things that make the family valuable in the first place and which are among the most important goods available to us as human beings.

Viewed from this perspective, as a harm reduction policy necessarily embedded in an unjust society, we see that the most powerful objections to parental licensing are quite different from those surveyed so far. Implementing parental licensing would involve granting authority to some state or other body to deny certain individuals the right to parent based on some test of competency, which will be devised by some government agency or private company and applied using individual judgement. Even granting that this would lead to the identification of some people who would go on to abuse children, thus reducing the incidence of child abuse, there are other very grave injustices and bad effects that would likely arise. In implementing parental licensing we would therefore take a huge risk that those who operate and manage the licensing system will themselves perform at a very high degree of competency and in good faith. This may require too much wishful thinking on the part of those who are in favour of parental licensing.

There are many examples of historical injustice involving the state making more than modest judgements about parental competency.^16^ These injustices have taken place in many different societies and have been very large in scale. In some cases, babies were illegally adopted and taken from unmarried mothers in Ireland and the UK.^17^ In other cases, such as in Australia, the racist, white supremacist policy of taking children from Australian Aboriginal and Torres Strait Islander families by government agencies and church missions into care institutions.^18^ White Australian mothers deemed “unfit” also had their children taken from them. In Canada, indigenous children were taken from their families and placed in residential schools, with the aim assimilation into “white culture”.^19^ Some were motivated by the need to separate children from mothers or parents deemed “unfit” to raise children, others had a more white supremacist motivation and involved mass separation of children born to indigenous people. In addition, we know that current policies of intervention in the family and denials of custody are racist and classist in their effects.^20^ Not only do these actions have devastating effects for the individuals involved, they entrench and reinforce discrimination against historically disadvantaged groups and some are arguably genocidal.^21^

Although it is possible to imagine a well-run licensing system that operates fairly, we should expect a licensing scheme that we adopt to have similar effects. In light of this, could such a system be justified? We have to weigh up the costs and the inequalities that are entrenched by such policies against the genuine prospect that children will avoid neglect and abuse. Of course, solving other social problems like racism and inequality, as well as poverty, should have a very high priority, but if a large enough group of children can be spared abuse, licensing may be justified.

We should also pay attention to how the licensing policy would be implemented as this brings to the fore further difficulties that could arise.^22^ Of course, ideally, everyone would get a license before they tried to procreate or adopt, and procreate or adopt only if they had a license or had reconciled themselves to not parenting the child. But that would almost certainly not be the case in all instances. There are broadly three ways to operationalise licensing and each give rise to objections.

- *(Reversible) Sterilisation*: sterilise everyone and only reverse the procedure for those who pass the test.
- *Adoption at Birth*: take children born to unlicensed parents into state custody. This would involve non-consensual physical separation of parents from their children.
- *Incentives*: incentivise test taking and voluntary adoption by those who fail the test.

All of the possible ways of implementing licensing are open to objections in addition to the violation of any right to parent. Sterilisation, even reversible, involves violations of rights to bodily integrity and the right to procreate, and amplifies the cost of discriminatory implementation of licensing. Adoption at birth can be highly traumatic for parents and would likely incentivize risky evasive measures being taken in late pregnancy, such as refraining from seeking medical help, that risk the wellbeing of the unborn child and the mother. Incentives may seem to offer a promising way out, and one that avoids rights violations of the kind that have been thought decisive against licensing. However, implementing an incentive scheme to get licensed would further disadvantage the poorest, who need the incentive the most, and so would subject themselves to testing that is inaccurate on false negative to a higher degree, while those who could afford not to get a license would not have to subject themselves to the risk of losing their children too.^23^

Further, there are issues around capacity for adoption. We have to think about how the children of unlicensed adults will be cared for. Ideally they would go to loving homes with licensed parents. However, it is likely that there will not be enough willing and licensed parents, not least because of how inaccurate the tests are. The alternative, then, is to place children in caring institutions which imposes its own harm and disadvantages and also decreases the chances that those already in those institutions will be adopted.^24^

These are the dimensions along which we need to think about parental licensing. We should resist the temptation to reject licensing wholesale because of the inaccuracy of testing or because it looks like it violates parental rights. Individually, these objections are insufficient. However, there are a whole host of reasons to be cautious or even sceptical about parental licensing based on its likely effects and likely implementation. When assessing parental licensing proposals, “Would it violate parental rights?” is not the only or even most important question. Instead we should focus on the more complex question “Is parental licensing part of the correct set of policies to respond to child abuse and neglect?” Answering this question requires us not only to consider the effects of any licensing proposal for the interests of competent parents, and the children, but also to consider licensing in combination with other policies, such as increased surveillance and parental education programs, and against the harms of entrenching disadvantage and prejudice and racism.^25^

## IV

In this paper, I have argued that two common objections to licensing depend on the existence of a stringent moral right to parent. I argued that such objections only succeed if we take these rights violations to be decisive objections to policies. I argued that this is implausible because it ignores the countervailing and very weighty, and most likely right-generating, interests of children in avoiding neglect and abuse. I suggested that this sort of objection is symptomatic of a deeper problem in the debate about parental licensing, in that critics and proponents have applied the wrong evaluative framework to the policy of licensing parenting. Instead of asking whether licensing violates rights, our assessment of the policy should proceed on the understanding that rights violations are inevitable and that applying the framework of harm reduction to the risky and harmful practice of parenting can shed light on the considerations relevant to its justification. If we adopt the harm reduction perspective, we can see that the most powerful reasons to be cautious and sceptical about parental licensing are to do with the state abuse of that power, and its likely discriminatory and other effects.

## Footnotes

1 LaFollette, Hugh. "Licensing parents." *Philosophy & Public Affairs* Vol. 9, No. 2 (1980): 182–197; Mangel, Claudia Pap. "Licensing parents: How feasible?." *Family law quarterly* (1988): 17–39 Vopat, Mark. "Parent licensing and the protection of children." In Samantha Brennan and Robert Noggle ed.s *Taking Responsibility for Children* (Wilfrid Laurier University Press, 2007): 73–96; Westman, Jack C. *Licensing parents: Can we prevent child abuse and neglect?*. Da Capo Press, 2007.

2 NSPCC, “Statistics on child abuse”, 19th February 2019. Accessed 19th January 2020: https://www.nspcc.org.uk/services-and-resources/research-and-resources/statistics/.

3 For an objection to La Follette’s argument see A. J. Cohen’s “The Harm Principle and Parental Licensing”, *Social Theory and Practice*, 43 (4) 2017, pp. 825–849.

4 Liao, S. Matthew. *The Right to be Loved,* Oxford University Press, 2015.

5 This could explain why licensing or vetting in the case of adoptive parents could be justified, but licensing biological parents could not. For further discussion of this point see Barry, Christian, and R. J. Leland. "Do Parental Licensing Schemes Violate the Rights of Biological Parents?." *Philosophy and Phenomenological Research* 94.3 (2017): 755–761 and Liao, S. Matthew. "Rightholding, Demandingness of Love, and Parental Licensing." *Philosophy and Phenomenological Research* 94.3 (2017): 762–769.

6 Wald, M. and Sandmire, M. “Licensing Parents – a response to Claudia Mangel’s proposal”, *Family Law Quarterly,* 24. 1 (1990): 53–76: 62. Because the prevalence rate of child abuse is so low, it is unlikely that a predictive instrument can be developed that will be able to identify a high proportion of future abusers without mislabeling a very large number of people as future abuser.

7 Ibid p. 59–60, and 68. See also footnote 14 for reference to other instruments.

8 One reason why inaccuracy of testing has been taken to be an objection to licensing, on its own, is that accuracy, or reliability, forms one of the conditions for analogy set out in La Follette’s argument for licensing. LaFollette, Hugh. "Licensing parents." *Philosophy & Public Affairs* (1980): 182–197:184.

9 See Wald and Sandmire, “Licensing parents”, fn. 11 on p. 56 for “To be more accurate, while many prediction instruments are reasonably good at selecting out of a general population those people who will, in fact, become abusers, they do so at the cost of mislabeling many people as likely abusers who will not, in fact, become abusers. As we discuss below, see notes 20–22 infra and accompanying text, there generally is a trade-off between being able to identify a high percentage of true abusers and the ability to limit the number of false identifications. In essence, the broader one casts one's nets to catch abusers, the more likely one is to catch all of them but at a cost of catching many other people who will not be abusers.” More minimalist licensing proposals have taking this point on board. See also, Vopat, M. “Parent licensing and the procreation of children.” I am minded to think that the concerns advanced by Wald and Sandmire apply here even since there is such a low base rate of abuse and neglect in society”.

10 It is possible that the right to parent is a will theory right instead, but most do not in fact defend it as such as that view is associated with implausible child-ownership positions so we need not consider it here.

11 Brighouse and Swift, *Family Values*, Princeton University Press, 2014, p. 95. In De Wispelaere, Jurgen, and Daniel Weinstock. “Licensing parents to protect our children?” *Ethics and Social Welfare* 6.2 (2012): 195–205: 199, the authors argue, borrowing Brighouse and Swift’s account of parental interests, that parenting is disanalogous to other licensed activities because of the weight of the interest they have in parenting and, in particular, the fact that there are no reasonable substitutes for it. They state that parenting “warrants special treatment” p. 198, because of this. This could be taken to be a parental right’s based objection to licensing.

12 For a critique of this way of characterizing the interest in parenting see Shields, Liam. "Parental rights and the importance of being parents." *Critical Review of International Social and Political Philosophy* 22.2 (2019): 119–133.

13 Scanlon, T. M., *What we owe to each other*. Harvard University Press, 1998. p. 235.

14 Infringing this right, as in the case of competent parents who would be denied the opportunity to parent by inaccurate licensing regimes, would call for some kind of redress. But it need not block licensing.

15 Some might argue that even denying incompetent parents the right to parent is objectionable since it is a case of pre-emptive punishment.

16 In Vopat, M. "Parent licensing and the protection of children." The author defends a minimal licensing scheme partly in response to some of these practical worries about the abuse of power and partly in response to worries about inaccuracy of testing. With respect to the abuse of power, however, I do not see how minimal licensing can avoid the problems. The transfer of power would still be enormous, as it ranges over every single parent–child relationship, even if it does so only to determine the meeting of a very low threshold. With respect to the inaccuracy of the testing, I do not see how large proportions of inaccurate on false negatives can be avoided, given the low rate of abuse in society.

17 See for example “Catholic church apologises for role in ‘forced adoptions’ over 30-year period”, *The Guardian*, 3rd November 2016. Accessed 20th January 2020: https://www.theguardian.com/society/2016/nov/03/catholic-church-apologises-for-role-in-forced-adoptions-over-30-year-period and “The lawyers seeking justice for survivors of Ireland’s force adoptions”, *Financial Times*, 4th July 2018. Accessed 20th January 2020: https://www.ft.com/content/f6d28a72-7ba6-11e8-bc55-50daf11b720d.

18 See for example, Australian Human Rights Commission, *Bringing them home: National Inquiry into the Separation of Aboriginal and Torres Strait Islander Children from Their Families*, 1997.

19 See, for example, Cassidy, Julie. "The Best Interests of the Child? The Stolen Generations in Canada and Australia." *Griffith Law Review* 15.1 2006: 111–152.

20 See for example Robert B. Hill, Casey-CSSP Alliance for racial equity in child welfare, an analysis of racial/ethics disproportionality and disparity at the national, state and county levels (2007). Available: https://www.citizenreviewpanelsny.org/documents/disproportionality_paper_bob_hill.pdf and Also, McMahon, Anthony, and Paula Allen-Meares. "Is social work racist? A content analysis of recent literature." *Social Work* 37.6 1992: 533–539. Nicole A Corley, Stephen M Young, Is Social Work *Still* Racist? A Content Analysis of Recent Literature, *Social Work*, 63.4, 2018: 317–326.

21 One might think that same issues of racial and other bias occur in the administration of other licenses, but that this is not sufficient reason to halt licensing those activities. On driving licenses see for example, https://www.theguardian.com/uk-news/2018/dec/03/black-women-far-less-likely-than-white-men-to-pass-driving-tests, accessed 9^th^ September 2020. However, there are many differences between these licensed activities and others, not least among them the profound loss for competent parents of custody of their child if denied a license. The loss of a driving license is not so profound. For a discussion of this contrast see De Wispelaere, Jurgen, and Daniel Weinstock. "Licensing parents to protect our children?." *Ethics and Social Welfare* 6.2 (2012): 195–205: 202.

22 I am grateful to Luara Ferracioli for highlighting this further set of problems.

23 Vopat M., "Parent licensing and the protection of children.": 82.

24 De Wispelaere and Weinstock, “Licensing parents to protect our children?”: 202.

25 Archard, David. "Child Abuse: parental rights and the interests of the child." *Journal of Applied Philosophy* 7.2 1990: 183–194; Bortolotti, Lisa, and Daniela Cutas. "Reproductive and parental autonomy: an argument for compulsory parental education." *Reproductive biomedicine online* 19 2009: 5–14.

